# The role of executive function in children's source monitoring with varying retrieval strategies

**DOI:** 10.3389/fpsyg.2014.00405

**Published:** 2014-05-08

**Authors:** Becky Earhart, Kim P. Roberts

**Affiliations:** Department of Psychology, Wilfrid Laurier UniversityWaterloo, ON, Canada

**Keywords:** executive function, cognitive development, source monitoring, working memory, inhibitory control

## Abstract

Previous research on the relationship between executive function and source monitoring in young children has been inconclusive, with studies finding conflicting results about whether working memory and inhibitory control are related to source-monitoring ability. In this study, the role of working memory and inhibitory control in recognition memory and source monitoring with two different retrieval strategies were examined. Children (*N* = 263) aged 4–8 participated in science activities with two sources. They were later given a recognition and source-monitoring test, and completed measures of working memory and inhibitory control. During the source-monitoring test, half of the participants were asked about sources serially (one after the other) whereas the other half of the children were asked about sources in parallel (considering both sources simultaneously). Results demonstrated that working memory was a predictor of source-monitoring accuracy in both conditions, but inhibitory control was only related to source accuracy in the parallel condition. When age was controlled these relationships were no longer significant, suggesting that a more general cognitive development factor is a stronger predictor of source monitoring than executive function alone. Interestingly, the children aged 4–6 years made more accurate source decisions in the parallel condition than in the serial condition. The older children (aged 7–8) were overall more accurate than the younger children, and their accuracy did not differ as a function of interview condition. Suggestions are provided to guide further research in this area that will clarify the diverse results of previous studies examining whether executive function is a cognitive prerequisite for effective source monitoring.

## Introduction

Between the ages of 3- and 8-years-old many fundamental changes in cognitive development occur rapidly, and one cognitive skill that shows drastic improvement is source monitoring (Roberts, 2002). The term “source” refers to the conditions under which a memory was acquired including many different attributes, such as when or where an event occurred or how it was perceived (e.g., whether it actually happened or was only imagined or seen on television; Johnson et al., 1993). Source monitoring is the process of making decisions about the source of a memory.

Although there is much research establishing that young children do not perform as well on source-monitoring tasks as older children or adults do, it is less clear why these age differences occur or what cognitive changes are supporting the development of source monitoring during early childhood. Thus, researchers are now addressing not only what recall strategies are most successful with young children, but also what underlying cognitive processes contribute to the development of source monitoring (e.g., Ruffman et al., 2001; Roberts and Powell, 2005; Kanakogi et al., 2012). Executive function is vital for many cognitive abilities, and this study addresses whether different aspects of executive function are necessary for the development of source monitoring in early childhood, or whether executive function is only more generally related to episodic memory. In particular, the ability to recall one source in the face of competing sources and to “compare and contrast” sources may be related to accurate source monitoring. The proposed executive function-source monitoring relationship was tested using two variations of a source-monitoring task: children were either asked to recall one source at a time *or* to consider two sources simultaneously in order to provide information about how executive function processes might contribute in somewhat different ways in each of these conditions.

### The source-monitoring framework

The Source-Monitoring Framework (Johnson et al., 1993) was developed to explain how source judgments are made. According to the framework, making attributions about the origin of a memory is a complex decision-making process that is more complicated than simple retrieval of source information because one can remember an event but not the circumstances under which the event occurred (e.g., who spoke, whether it was a dream). Source decisions are often based on the qualitative characteristics of memory traces, such as the spatial or temporal context, the amount of perceptual detail, the cognitive operations associated with the memory, semantic details, and the affective response when the memory was formed. Critically, memories originating from the self (e.g., dreams, own actions, thinking through a plan) comprise a different qualitative profile than memories of external sources such as a colleague describing an issue to you or watching a movie. Typically memories of internal (self-generated) events contain fewer perceptual details and more cognitive operations than memories of events derived external to an individual; the profile of externally-derived memories is the reverse of internal sources. For example, when distinguishing between an event that actually happened and something that was only imagined, it could be expected that a real event would have greater perceptual detail and fewer records of cognitive operations than an imagined event.

Source decisions are made either through heuristic or systematic judgment processes. Heuristic processes involve quick decisions that may occur in the course of remembering without conscious awareness of making a decision (e.g., remembering the source of a memory because you recalled it in the person's voice; Johnson et al., 1993). Systematic judgment processes are more analytic and deliberate; when making systematic decisions, a person will reason carefully about what is possible given the information that they have from the memory itself. This may involve retrieving supporting memories, reasoning about constraints, and employing strategies (Johnson et al., 1993).

### The development of source monitoring

As mentioned earlier, extensive past research on source monitoring has clearly established that young children (aged 3–4 years) have substantially poorer source-monitoring abilities than do older children (e.g., 8–12 year-olds) or adults (e.g., Gopnik and Graf, 1988; Lindsay et al., 1991; Powell and Thomson, 1996; Roberts and Blades, 1999). It is not until approximately age 10 that children perform as well as adults on many source-monitoring tasks (see Roberts, 2002, for a review). Given that the quality of the memory and the quality of the decision-making process are both important components of accurate source monitoring (Johnson et al., 1993), young children may not have the necessary cognitive prerequisites to engage in systematic processing. For example, young children may not be able to coordinate the many decision-making processes necessary for systematic processing, and therefore cannot reason about the constraints of memories in order to problem-solve. The maturational development of the frontal lobe may have to be in motion before complex and effortful decisions can be made (De Luca and Leventer, 2008).

### Executive function and source-monitoring decisions

Research on adults with frontal lobe damage demonstrates that these participants show deficits in memory and fail to monitor the sources of their memories effectively. Adults with frontal lobe damage show many of the same problems that young children demonstrate in source monitoring, suggesting that the frontal lobe is implicated in the development of source monitoring (Schacter et al., 1995). Differences in executive function, a broad category of skills that support goal-directed behavior, have been linked with immature frontal lobe development (e.g., De Luca and Leventer, 2008). Executive function underlies many cognitive abilities, and can potentially be linked with skills required for source monitoring.

There are theoretical reasons to believe that two components of executive function, inhibitory control and working memory, would be related to source-monitoring accuracy. Inhibitory control might be related because source monitoring requires inhibition of the familiarity-based retrieval processes that are often used automatically to make recognition decisions (Ruffman et al., 2001), as well as inhibition of information from competing sources. Working memory may be related to source monitoring because it is involved in controlling attention, and therefore plays a role in designating what information cognitive resources will be allotted to (Gerrie and Garry, 2007). A complex process of reasoning about the constraints of memories, retrieving supporting memories, comparing and contrasting sources, and inhibiting competing information may be needed to make effective decisions about source.

Research in this area has looked for links between executive function and source monitoring, as well as susceptibility to suggestibility and false memories (which are source-monitoring errors in which people fail to recognize the source of information that was suggested to them). A study examining this relationship in an elderly population found that those who had high composite scores on a battery of executive function tests were better at a source-monitoring task involving voice identification than a group who scored low on the executive function tests (Glisky et al., 1995). This suggests that impairments in executive function may partially account for source-monitoring decline in old age.

Research with younger adults has shown connections between working memory and resistance to misleading suggestions. Using a misinformation paradigm where participants were exposed to misleading post-event information, Jaschinski and Wentura (2002) found that adults with a higher working memory capacity were misled to a lesser extent by misinformation than adults with a lower working memory capacity. Gerrie and Garry (2007) replicated these findings and added that the effect of working memory capacity was especially strong for crucial event details as opposed to non-crucial, or peripheral, details. Researchers have suggested that working memory is negatively related to susceptibility to false memories because people with higher working memory capacities are better able to monitor the sources of information (Gerrie and Garry, 2007), and this was supported by a study that showed source monitoring as a mediator between working memory and false recall (Unsworth and Brewer, 2010).

Studies addressing the relationship between executive function and source monitoring in children are more informative about whether executive function underlies developmental improvements in source-monitoring accuracy. However, studies specifically examining this relationship with children are not extensive, and have contradictory results. Several studies have shown that executive function is not predictive of source-monitoring accuracy in children. For example, one study found that cognitive shifting predicted source monitoring, but inhibitory control did not (Kanakogi et al., 2012). In a comprehensive review of individual difference factors in suggestibility, Bruck and Melnyk (2004) reported that there was typically a negative relationship between executive function and suggestibility, but that few studies showed significant correlations.

Other studies, on the other hand, have supported the role of executive function in source-monitoring development. Roberts and Powell (2005) found that children with better inhibitory control were less suggestible to misleading information, and Karpinski and Scullin (2009) found that preschoolers with better executive function were less suggestible in a pressured suggestive interview. The latter study showed significant relationships for both inhibitory control and working memory.

Melinder et al. (2006) found mixed results, with inhibitory control as a significant predictor of suggestibility, but not source monitoring. Similarly, Ruffman et al. (2001) have found a relationship between inhibitory control and source accuracy, but only for some types of source-monitoring questions. They did, however, find clear evidence that working memory was related to source monitoring. Overall, the results of this literature are inconclusive with some researchers finding significant relationships and others finding no evidence of the role of executive function.

In addition, several studies finding significant relationships between suggestibility or source monitoring and executive function have used memory tasks that load heavily on both recognition and source memory. For example, in a typical suggestibility paradigm, children are asked whether or not suggested details occurred during a real event; this requires a simultaneous assessment of whether the child recognizes the detail, as well as its source (real or suggested; e.g., Roberts and Powell, 2005; Karpinski and Scullin, 2009). Similarly, in source-monitoring studies children may be asked whether details occurred in source A, source B, or neither source. Because children are also asked about things that happened in neither source, they must assess both if the detail is familiar, and if so, which source it is from (e.g., Foley et al., 1983; Foley and Johnson, 1985; Ruffman et al., 2001). In these studies, then, it is unclear whether executive function is significantly related to recognition memory, source monitoring, or both.

The present study seeks to add to the body of literature on the relationship between executive function and children's developing source-monitoring skills to test whether executive function is an important predictor of source-monitoring accuracy, or whether executive function plays a more general role in episodic memory after exposure to multiple sources. Our procedure separated recognition and source-monitoring tasks with a two-step test; first children identified the details they had seen, and then they made source decisions about them. With this procedure we sought to look at the relationships of executive function to recognition memory and source monitoring separately, to determine whether executive function makes a unique contribution to source monitoring when recognition memory demands are removed.

### Serial vs. parallel recall of sources

The cognitive abilities involved in source monitoring may differ depending on retrieval strategy. In previous studies of source monitoring children have been asked about sources serially (one source at a time, reporting everything they remember about one source, followed by everything they remember about another source; Thierry et al., 2001, 2005) or in parallel (questioned about multiple sources of information simultaneously; Powell and Thomson, 2003). The role of executive function in source monitoring may vary depending on the way the task is structured.

If children are asked to consider sources serially, they are required to inhibit the reporting of information from other irrelevant sources, including sources within the same event (Roberts and Powell, 2005). Consequently, inhibitory control may play a stronger role in source monitoring when the task involves reporting information about one source at a time. On the other hand, when children are asked to consider sources in parallel, they must hold information about the characteristics of multiple sources in working memory in order to compare and contrast them. When asked about multiple sources at the same time working memory may play a more important role in source-monitoring ability. Therefore, this study also tested whether inhibitory control and working memory would be differentially related to source-monitoring accuracy when children are questioned about sources serially vs. in parallel, because recalling information from one source while holding back information from other sources may require different cognitive skills than comparing and contrasting competing sources.

Additionally, different retrieval strategies may lead to differences in source-monitoring accuracy. Source judgments are often based on comparing the relative strength of characteristics (e.g., the amount of perceptual detail) to determine which source “fits” better with a memory (Johnson et al., 1993). The Source-Monitoring Framework might predict that source decisions would be more accurate when considering multiple sources at the same time, because thinking about different sources at the same time would enable a more direct comparison of relevant characteristics than thinking about sources in a serial fashion. This could lead to higher source-monitoring accuracy compared to asking about sources serially, when a strategy is not facilitated. If asked about sources serially, children would be required to spontaneously generate the comparison strategy in order to source monitor with similar accuracy levels. A third goal of this study was to empirically test the prediction that source-monitoring accuracy would be higher for children who were asked about sources in parallel than those asked about sources serially.

### The present study

This study examined the relationship between executive function, recognition memory and source monitoring both generally and with two different retrieval strategies (serial vs. parallel). Children aged 4–8 participated in science activities interactively and by listening to a story (i.e., the target sources). The children were given a recognition and source-monitoring test after four to seven days. During a third session, children's working memory and inhibitory control were measured to determine whether these cognitive variables were related to source monitoring, and whether the role of these two variables differed for children in the serial and parallel conditions. Following from the discussion above, this study had four hypotheses:

*Hypothesis 1:* Age differences were expected in executive function, recognition memory and source monitoring, consistent with the large body of literature demonstrating development across childhood in all of these areas. Though this hypothesis was not novel, we wanted to confirm that there were in fact age differences in both executive function and memory before attempting to examine relationships between them.

*Hypothesis 2:* It was expected that working memory and inhibitory control would be significant predictors of both recognition and source memory.

*Hypothesis 3:* An interaction was predicted such that working memory would be a stronger predictor of source accuracy in the parallel condition, whereas inhibitory control would be a stronger predictor of source accuracy in the serial condition.

*Hypothesis 4:* Overall differences in source-monitoring accuracy between the serial and parallel interview conditions were predicted, with children making more accurate source decisions when considering sources in parallel than when considering sources serially.

## Methods and materials

### Design

This study had a 3 (Age in years: 4–5, 6, 7–8) × 2 (Interview Condition: Serial, Parallel) × 2 (Source Presentation: Real-Life, Story) mixed design with the last factor within-subjects.

### Participants

Initially 308 children from local daycares, elementary schools, and a university summer day camp participated. Eighteen participants did not complete the study because they missed a session and an additional 27 who completed the study were excluded [12 did not provide any details in free recall, indicating that they did not remember the activities; 3 showed evidence of a yes bias (i.e., a response bias toward saying yes they recognized every detail, including misleading ones) and 12 were excluded due to interviewer errors (e.g., asking source questions about details the child said were not present at the activities)]. The excluded participants were equally distributed across age groups and interview conditions.

The final sample was 263 4- to 8-year-old children (52% male). Four participants did not complete the executive function tests due to time constraints during testing and therefore were excluded from analyses of the cognitive variables, but their memory scores were still included in source accuracy comparisons between age groups and interview conditions. The 4- to 5-year-olds (*n* = 84, 41 in the serial condition) had a mean age of 5.04 (*SD* = 0.59), the 6-year-olds (*n* = 79, 40 in the serial condition) had a mean age of 6.46 (*SD* = 0.28) and the 7- to 8-year-olds (*n* = 100, 49 in the serial condition) had a mean age of 7.87 (*SD* = 0.59). The children were recruited from a mid-sized Canadian city. Information about participants' ethnicity was not available, but the majority of participants were Caucasian and from middle-class families. Informed consent was obtained from a parent/guardian prior to the beginning of data collection, and children assented to participate. There was no monetary compensation for participation. Participants were randomly assigned to one of two interview conditions with the constraint that there were approximately equal numbers of children from each age group and gender in each interview condition.

### Materials and procedure

#### Event

Groups of up to 10 children participated in science activities about the human body comprising an interactive activity referred to as the “real-life demonstration” and a story. There were two presentation scripts and they were counterbalanced so that each was shown as the real-life demonstration half of the time and the story the other half of the time. The order of the presentations was also counterbalanced. The presentations each lasted approximately 10 min and had similar content (i.e., a researcher using simple experiments and science materials to teach children about the body) but the research assistant that conducted the demonstration and the main character of the story were different people. The story was a PowerPoint presentation with text and photos presented on a laptop.

The sources were clearly labeled for the children throughout the event by repeatedly referring to them as “the real-life demonstration” and “the story.” In each presentation there were 12 details that would be tested during the memory interview, and these details were highlighted during the presentations to ensure that children paid attention to and encoded them.

#### Baseline memory test

A baseline memory assessment was administered immediately after the event to measure encoding. The relationship between this measure of recognition memory after no delay and executive function was tested, but this measure also served to ensure that there were no differences between interview conditions in initial event memory. The test included 10 recognition questions about event details that were not included in the later memory interview (five from the real-life demonstration and five from the story). The questions were asked in random order, with no reference to the source of the details. Accuracy proportions were calculated for analyses by dividing the number of correct answers by the number of questions asked.

#### Memory interview

After four to seven days children were interviewed individually by a new research assistant who was blind to counterbalancing condition and therefore was not aware of whether the children were correct or not when choosing the source of details. At the beginning of the 30-min interview, the interviewer introduced herself and spent a few minutes building rapport with the child. The children were given the chance to freely recall anything they could remember about the activities in response to open-ended prompts about what happened, confirming that they remembered the activities and both sources. When children had reported everything they could remember, the interviewer continued to the recognition and source questions.

The memory test was a modified version of the posting-box procedure (Bright-Paul et al., 2005). Participants were required to sort cards depicting details into boxes that represented the sources of the details. Ideally, children would have high hit rates so that they could make source judgments about many event details that they experienced. As well, for source-monitoring accuracy, the ideal level of task difficulty would be such that children were performing above chance but not at ceiling in order that the sample would have enough variability to be related to other variables. This procedure yielded optimal results with children doing well on source monitoring, but not performing at ceiling in any age group (see the Results section Source Accuracy for further details).

There were 36 photographs (3 × 4 inches) comprising the 12 non-misleading details from each source and 12 misleading details that were not presented in either source. Children in both conditions first completed a *recognition task*. They were asked to place pictures of details that they remembered from the event in a “Yes” box, and pictures they did not remember from the event in a “No” box (a “Don't Know” box was also available). The cards were shuffled and shown to children one at a time as the interviewer asked about the details (e.g., “Did you ever see *dirt from the garden* at the activities?”). Once children had sorted all 36 cards, the interviewer took cards placed in the “Don't Know” box and gave children a second opportunity to sort through those cards before asking source questions.

The subsequent *source-monitoring task* began by retrieving the cards from the “Yes” box (i.e., details children claimed were in the event). Children were asked to sort the cards into three different boxes to indicate their source: “Real-Life Demonstration,” “Story,” and “Don't Know.” However, these boxes were presented differently to children in the serial and parallel conditions.

For the children in the *serial condition*, the interviewer presented one box at a time (order was counterbalanced) and thus, children were required to consider the sources one after the other. Cards were laid out four at a time and the interviewer provided a label for each picture. The children were asked to look through the cards carefully and put any pictures from the story (for those children with the story box first) in the “Story” box. The remaining cards were set aside. After going through all the cards, the interviewer presented the children with the other source box, and the children went through the leftover cards again, now considering the second source (e.g., the “Real-Life Demonstration” box). Any cards that were not attributed to either source were recorded as “Don't Know.”

For the children in the *parallel condition*, the interviewer brought out the “Story” box, the “Real-Life Demonstration” box and the “Don't Know” box at the same time. The interviewer showed the children cards one at a time and labeled the picture, and as the children considered each detail they decided if it belonged in the “Story” box or the “Real-Life Demonstration” box (or the “Don't Know” box if they were unsure about the source). In this condition, the children considered both sources as they thought about where they saw each detail because they had to decide whether it came from the story or the real-life demonstration. After completing the interview, children were thanked for their participation and brought back to their classrooms.

Proportions were calculated for hits (correct identification of non-misleading details), false alarms (incorrect identification of misleading details as having been present at the activities) and source accuracy for story details and real-life details separately. A recognition accuracy score was then calculated by subtracting the proportion of false alarms from the proportion of hits. “Don't know” responses were conservatively coded as incorrect for both recognition and source scores. Scores were summed by two independent coders to prevent errors. The nature of the coding was very objective (i.e., counting correct responses), so inter-rater reliability was greater than 99%. The few disagreements were due to addition errors and were resolved before data analysis.

#### Cognitive assessments

Within approximately one week of the interview, participants completed a third session individually for approximately 15 min. Children were given a battery of cognitive tests consisting of two working memory tasks and two inhibitory control tasks. These tasks were presented as games to the children.

***Working memory***. The working memory tests were from the WISC-IV Digit Span subtest (Wechsler, 2003). In the Forward Digit Span test, the participant heard a sequence of numbers and was asked to repeat the sequence. The first trial began with a sequence of two digits and the sequences got progressively longer, up to a maximum of nine digits. There were two trials for each sequence length, and after successful repetition of at least one of those sequences, the sequence length increased by one digit. Testing continued until the participant failed both trials of a sequence length. Children were scored one point for each correct repetition for a maximum score of 16.

The Backward Digit Span test was conducted similarly to the Forward Digit Span test, but in this task participants heard a sequence of numbers and had to repeat the sequence in backwards order. Again, the test began with sequences of two digits and the sequences increased in length by one digit every two trials. The maximum number of digits in a sequence was eight. Participants were given one example and one practice trial before testing commenced. If they answered the practice trial correctly, testing began and continued until the participants incorrectly answered both trials of a sequence length. If the participants did not answer the practice trial correctly, they were given up to two more practice trials. If they still could not answer correctly, testing was discontinued. Children were scored one point for each correct repetition for a maximum score of 16.

***Inhibitory control***. Participants completed two measures of inhibitory control that have frequently been used in previous literature and are easy to administer: Luria's Hand Game (e.g., Hughes, 1996; Fahie and Symons, 2003) and the Day/Night Stroop task (e.g., Gerstadt et al., 1994; Reck and Hund, 2011). In Luria's Hand Game, the researcher either pointed a finger or made a fist, and the child was asked to make the opposite hand gesture from what the researcher did (e.g., make a fist when she pointed a finger). There were 20 trials in one randomized order: “Fist, Finger, Finger, Fist, Fist, Finger, Finger, Fist, Finger, Finger, Fist, Fist, Fist, Finger, Fist, Finger, Finger, Fist, Fist, Finger.” Children were encouraged to respond as quickly as they could. Participants were given a practice trial of each gesture before beginning. On each trial participants were scored one point if they produced the opposite hand gesture or immediately self-corrected their action. A score out of 20 was computed based on the number of successful trials.

The Day/Night Stroop task is a modified Stroop task for children that involves looking at pictures of day and night and saying the opposite of what the picture represents. The pictures were shown in a PowerPoint presentation on a laptop. These pictures are universally recognizable, even for young children: the “day” picture was a blue sky with a sun and clouds, and the “night” picture was a black sky with a moon and stars. Participants were encouraged to respond as quickly as possible, and the slide was advanced to a new picture as soon as they responded. There were 20 trials in one randomized order: “Night, Day, Night, Night, Day, Night, Day, Day, Night, Day, Day, Night, Night, Day, Night, Day, Day, Night, Day, Night.” Children were scored one point for each trial where they said the opposite of what was shown (e.g., saying “day” when shown the picture of a moon), and a score out of 20 was calculated.

The order of the four tests was randomized with the constraint that participants received the Forward Digit Span task prior to the Backward Digit Span task.

## Results

### Analytic strategy

Analyses were conducted to first explore developmental differences in memory accuracy and executive function, as well as differences in source accuracy between interview conditions. We then analyzed the relationship between memory accuracy and executive function, and whether there was an interaction with interview condition. An alpha level of 0.05 was used to determine significance for all analyses, unless otherwise noted.

### Preliminary analyses

Preliminary analyses confirmed that there were no overall differences between interview conditions in age, delay measured in days, baseline memory scores, working memory scores, inhibitory control scores, the number of details freely recalled at the beginning of the interview, or recognition accuracy during the memory test (all *t*s ≤ 1.57, all *p*s ≥ 0.12). There were also no differences between age groups in delay, *F*_(2, 260)_ = 2.17, *p* = 0.12.

All four cognitive scores (two inhibitory control scores and two working memory scores) were significantly correlated with each other, *r*s ≥ 0.20, *p*s < 0.001, and when age was controlled the results were similar (although the magnitude of the correlations were smaller); see Table 1 for the correlations. Although the correlations between the two working memory scores and between the two inhibitory control scores were significant, the magnitude of the correlations did not justify combining the measures into two composite scores. Therefore, analyses were conducted on all four cognitive variables.

**Table 1:** **Correlations between scores on the inhibitory control tasks and working memory tasks**.

***n* = 259**	**1**	**2**	**3**	**4**
1. Day/night stroop	–	0.25^**^	0.23^**^	0.42^**^
2. Luria's hand game	0.22^**^	–	0.24^**^	0.20^**^
3. WISC digit forward score	0.08	0.21^**^	–	0.51^**^
4. WISC digit backward score	0.28^**^	0.15^*^	0.35^**^	–

*Partial correlations controlling for age are shown in the bottom half of the table*.

* *Significant at the 0.05 level (2-tailed)*.

** *Significant at the 0.01 level (2-tailed)*.

### Developmental and interview condition differences

#### Executive function

The inhibitory control measures showed some evidence of a ceiling effect. There was enough variability, however, to find significant correlations with other variables (see below). The working memory scores showed more variability. The means and standard deviations for the four measures by age group are displayed in Table 2. Because all of the cognitive variables were correlated, a one-way multiple analysis of variance (MANOVA) was used to compare the scores from three age groups on all four cognitive variables. There was a significant multivariate effect [Wilk's λ = 0.68, *F*_(8, 506)_ = 13.60, *p* < 0.001, η^2^_*p*_ = 0.18]. Follow-up 3 (Age) One-Way analyses of variance (ANOVAs) showed age differences for all variables (*F*s ≥ 2.43, *p*s ≤ 0.04, one-tailed). *Post-hoc* Bonferroni comparisons for the inhibitory control tasks showed that 4- to 5-year-olds had lower inhibitory control scores than 6-year-olds or 7- to 8-year-olds, but the older age groups did not differ. Bonferroni comparisons for the working memory variables showed that all three age groups were different from each other on both measures, demonstrating significant improvements in working memory for each age group.

**Table 2:** **Mean number of accurate responses for executive function measures by age group**.

**Age group (years)**	**Inhibitory control tasks**		**Working memory tasks**	
	**Luria's hand game (maximum 20)**	**Day/night stroop (maximum 20)**	**WISC digit span forward (maximum 16)**	**WISC digit span backward (maximum 16)**
4–5	15.60	16.62	6.12	3.53
(*n* = 81)	(2.71)	(3.50)	(1.71)	(1.91)
6	16.39	18.33	7.13	5.00
(*n* = 79)	(2.33)	(2.09)	(1.70)	(1.50)
7–8	16.22	18.63	7.89	5.80
(*n* = 99)	(2.16)	(1.66)	(1.48)	(1.29)
Total	16.08	17.90	7.10	4.85
(*n* = 259)	(2.41)	(2.64)	(1.78)	(1.83)

*Standard deviations are in parentheses*.

#### Recognition accuracy

Across age groups, the proportion of accurate responses (hits and correct rejections) had a mean of 0.81 (*SD* = 0.10), and ranged from 0.47 to 1.00. Recognition scores calculated by subtracting false alarms from hits were subjected to a 3 (Age in years: 4–5, 6, 7–8) One-Way ANOVA to determine whether there were age differences. There was a main effect of age, *F*_(2, 260)_ = 24.06, *p* < 0.001, η^2^_*p*_ = 0.16, and Bonferroni *post-hoc* comparisons revealed that all three age groups differed from each other (*M*_4−5_ = 0.57, *SD* = 0.21; *M*_6_ = 0.64, *SD* = 0.19; *M*_7−8_ = 0.75, *SD* = 0.14), demonstrating a steady improvement in recognition memory with age.

#### Source accuracy

The mean source accuracy proportion was 0.71 (*SD* = 0.18), and scores ranged from 0 to 1.00. A 3 (Age: 4–5, 6, 7–8) × 2 (Interview Condition: Serial, Parallel) × 2 (Source Presentation: Real-Life, Story) analysis of covariance (ANCOVA) was run with repeated measures on the last factor to evaluate hypotheses 1 and 4: whether there were developmental differences and/or interview condition differences in source accuracy. The baseline accuracy proportion was included as a covariate because the baseline scores were correlated with age, and it was significant, *F*_(1, 256)_ = 17.85, *p* < 0.001, η^2^_*p*_ = 0.065.

The analysis revealed a main effect of age *F*_(2, 256)_ = 5.18, *p* = 0.006, η^2^_*p*_ = 0.039, confirming developmental differences in source accuracy. Bonferroni *post-hoc* comparisons showed that the 4- to 5-year-olds (*M* = 0.66, *SD* = 0.22) made fewer accurate source judgments than the 6-year-olds (*M* = 0.74, *SD* = 0.17) or 7- to 8-year-olds (*M* = 0.79, *SD* = 0.14), who did not differ from each other. Even the youngest age group performed above chance (0.50), *t*_(83)_ = 6.43, *p* < 0.001.

There was also a main effect of interview condition, *F*_(1, 256)_ = 25.72, *p* < 0.001, η^2^_*p*_ = 0.091 and an age by condition interaction, *F*_(2, 256)_ = 4.50, *p* = 0.01, η^2^_*p*_ = 0.034. Children in the parallel condition (*M* = 0.76, *SD* = 0.16) were more accurate than those in the serial condition (*M* = 0.66, *SD* = 0.20). Follow-up *t*-tests comparing the accuracy scores of children in the serial and parallel conditions within each age group revealed that the condition effect was significant for the 4- to 5-year-old and 6-year-old age groups, *t*s ≥ −2.77, *p*s ≤ 0.007, but not for the 7- to 8-year-olds, *t*_(98)_ = −0.99, *p* = 0.32.

Finally, there was a main effect of source presentation, *F*_(1, 256)_ = 6.10, *p* = 0.01, η^2^_*p*_ = 0.023, but no interactions involving source presentation, *F*s ≤ 0.44, *p*s ≥ 0.60. Children made more accurate source judgments about details from the real-life demonstration (*M* = 0.83, *SD* = 0.23) than about details from the story (*M* = 0.59, *SD* = 0.30). See Table 3 for the mean source accuracy scores by age group, source presentation and interview condition.

**Table 3:** **Mean source accuracy proportions by age, interview condition and source presentation**.

**Age group**	**Interview condition**	**Source presentation**	
		**Real-life**	**Story**
4–5	Serial	0.66	0.45
	(*n* = 41)	(0.35)	(0.35)
	Parallel	0.80	0.60
	(*n* = 43)	(0.21)	(0.28)
6	Serial	0.78	0.54
	(*n* = 40)	(0.25)	(0.29)
	Parallel	0.92	0.66
	(*n* = 39)	(0.16)	(0.27)
7–8	Serial	0.89	0.64
	(*n* = 49)	(0.15)	(0.29)
	Parallel	0.93	0.65
	(*n* = 51)	(0.08)	(0.28)

*Standard deviations are in parentheses*.

### The relationship between executive function and memory variables

#### The relationship between executive function and memory in the overall sample

As a first step to explore the contribution of executive function in memory and source monitoring, correlations were run among the cognitive variables and all memory tasks in the study. All four of the cognitive variables were significantly correlated with baseline, recognition, and source accuracy scores (*r*s ranging from 0.13 to 0.41), except that Luria's Hand Game was not correlated with source accuracy, *r*_(257)_ = 0.08, *p* = 0.18 (see Table 4 for the full set of correlations). Overall there was evidence that the cognitive variables were related to both recognition and source accuracy. However, when partial correlations were run controlling for age, only Stroop and WISC Backward scores were related to baseline and recognition accuracy, and none of the tasks were related to source accuracy.

**Table 4:** **Correlations between memory scores and executive function scores**.

***n* = 259**	**Inhibitory control tasks**		**Working memory tasks**	
**Memory scores**	**Luria's hand game**	**Day/night stroop**	**WISC digit forward**	**WISC digit backward**
Baseline accuracy	0.14^*^	0.35^**^	0.31^**^	0.41^**^
	(0.09)	(0.22^**^)	(0.11)	(0.18^**^)
Recognition accuracy	0.13^*^	0.25^**^	0.23^**^	0.35^**^
	(0.09)	(0.12)	(0.05)	(0.15^*^)
Source accuracy	0.08	0.16^**^	0.23^**^	0.27^**^
	(0.04)	(0.05)	(0.10)	(0.10)

*Partial correlations controlling for age are in parentheses*.

* *Significant at the 0.05 level (2-tailed)*.

** *Significant at the 0.01 level (2-tailed)*.

Linear regression analyses were run to determine whether executive function scores were predictive of baseline, recognition, and source accuracy scores. All four cognitive scores were entered as predictors simultaneously. For baseline accuracy proportion, Luria's Hand Game did not significantly contribute to the variance in the model, but Stroop scores and both WISC Digit Span scores were significant predictors. Therefore, both inhibitory control and working memory were predictive of memory for the event details immediately afterwards. The model accounted for 22% of the variance in baseline memory. When age was added as a predictor only the Stroop scores remained significant. Standardized regression coefficients and their associated test statistics for significant predictors can be found in Table 5 for all regression analyses reported.

**Table 5:** **Standardized regression coefficients and test statistics for significant and marginal predictors in regression analyses**.

**Dependent variable**	**Predictor**	**Beta**	***t***	***p***	***p* when age is entered**
Baseline accuracy	Day/night stroop	0.21	3.45	0.001	0.007
	WISC digit span forward	0.13	2.02	0.04	0.40
	WISC digit span backward	0.25	3.68	<0.001	0.11
Recognition accuracy	Day/night stroop^Δ^	0.12	1.81	0.07	0.26
	WISC digit span backward	0.26	3.59	<0.001	0.08
Source accuracy	WISC digit span forward^Δ^	0.13	1.79	0.07	0.31
	WISC digit span backward	0.18	2.36	0.02	0.31

Δ *indicates marginal significance level*.

Only one score emerged as a significant and independent predictor of recognition accuracy: the WISC Backward Digit Span. The other three tests did not reach significance, although the significance level for the Stroop task was marginal. The model accounted for 14% of the total variance in recognition accuracy. There was evidence for the role of working memory, but when age was entered into the regression, the WISC Backward scores were only marginally significant as a predictor.

For source accuracy scores, there were significant effects of working memory, but not of the inhibitory control variables. Working memory scores explained 9% of the variance in source accuracy scores. When age was entered into the regression, no significant predictors except for age remained.

#### The relationship between executive function and source accuracy as a function of retrieval strategy

The relationship between working memory and inhibitory control in the serial and parallel conditions was examined. To do so, separate correlations were run between the source scores of children in the serial and parallel conditions and the executive function measures, across age. All correlations for both conditions can be found in Table 6. For children in the serial condition, there were no correlations between the inhibitory control measures and source accuracy scores (*r*s ≤ 0.12, *p*s ≥ 0.19), but both working memory scores were significantly correlated with source accuracy. When these correlations were rerun as partial correlations controlling for age, only the WISC Backward scores were marginally related.

**Table 6:** **Correlations between executive function scores and source accuracy in the serial and parallel interview conditions**.

**Interview condition**	**Inhibitory control tasks**		**Working memory tasks**	
	**Luria's hand game**	**Day/night stroop**	**WISC digit forward**	**WISC digit backward**
Serial	0.04	0.12	0.31^**^	0.31^**^
	(−0.01)	(−0.01)	(0.15)	(0.10)
Parallel	0.11	0.23^**^	0.16	0.27^**^
	(0.08)	(0.14)	(0.05)	(0.15)

*Partial correlations controlling for age are in parentheses*.

*Significant at the 0.05 level (2-tailed).

** *Significant at the 0.01 level (2-tailed)*.

For children in the parallel condition, source accuracy scores were correlated with scores on the Stroop task and scores on the Digit Span Backward test, but not with Luria's Hand Game or Forward Digit Span scores. When age was controlled, these relationships were no longer significant (*p* = 0.11 and 0.10 for Stroop and Digit Span Backward, respectively).

## Discussion

The main purpose of this study was to evaluate the role of executive function as a predictor of episodic memory after exposure to multiple sources and of source-monitoring ability. In one condition, source monitoring was facilitated for the children by asking them to compare the two different sources (“real-life demonstration” and “story”); other children were asked about sources serially (i.e., they recalled details from one source first, and afterwards the other source). We expected that measures of working memory and inhibitory control would be related to both recognition and source accuracy, but that these relationships might be different in the serial and parallel conditions. Additionally, we expected that source decisions would be more accurate when details from the sources were recalled in parallel than when they were recalled serially.

### Developmental differences

We wanted to verify that, consistent with a large body of literature showing developmental changes between ages 4 and 8, there would be improvements in recognition, source monitoring and executive function; indeed we did find such patterns. While this is a replication of the majority of the findings in this area, it was important to establish that the pattern was the same in our particular source-monitoring tasks. Increases in accuracy with age provided the necessary data to test for relations between executive function and source monitoring. Importantly, all age groups performed well at identifying the details from the event, and all scored above chance on the source-monitoring task. Thus, the children in our sample were genuinely remembering the details and trying to identify their sources.

There was also an effect of source presentation. Source accuracy scores were better for the real-life demonstration than the story, indicating that this source was more salient for the children. In addition, children showed a “real-life bias”; that is, a bias toward reporting that details had come from the real-life demonstration more often. This is evidence of familiarity-based processing, because children reasoned that if they remembered seeing a detail, it must have happened in “real-life.”

### Memory, source monitoring, and executive function

Two components of executive function were examined, and there was support for the hypothesis that both recognition and source monitoring are significantly correlated with measures of working memory and inhibitory control. Higher executive function scores were associated with better initial memory for the event, better delayed recognition of the details, and better identification of the sources. Regression analyses revealed that both working memory and inhibitory control were predictive of memory for event details immediately after the event and after a delay, but only working memory predicted source accuracy.

Generally, the significant relationships we found were weaker or non-significant when age was controlled for, which suggests that although executive function is related to recognition and source monitoring, a more general “cognitive development” factor is a stronger predictor than executive function alone. Clearly there are relationships between executive function, especially working memory, and source monitoring, as well as between executive function and recognition memory both immediately and after a delay. However, age as a construct represents improvements in many developmental processes, including theory of mind and reasoning about conflicting mental representations, which have also been shown to account for variance in source monitoring (Welch-Ross et al., 1997; Welch-Ross, 1999; Bright-Paul et al., 2008). Because age is tied to executive function as well as other cognitive abilities that are important for source monitoring, it is of course a stronger predictor than executive function alone.

The relationship between executive function and baseline accuracy scores suggests that executive function may play a role not only at retrieval, but also at encoding; those children with higher executive function scores recalled more accurate information about the event when there was no delay, and hence very little forgetting. Source monitoring may be enhanced by this initial processing because it could also be necessary at encoding to bind together features that allow source to be encoded with the memory, or enough suitable information to reason about source later. This is consistent with an argument from a recent review by Mammarella and Fairfield (2008) that working memory is important at encoding for binding the features of events together, which is crucial for source monitoring.

We hypothesized that working memory would be related to source-monitoring accuracy and this was supported. We reasoned that working memory may be necessary for holding information about different sources in mind, and engaging in “compare and contrast” reasoning. That is, if the two sources (live event and story) were recalled by the children, they would need to compare these sources with each other in order to decide the correct source. The results are similar to Ruffman et al.'s (2001) work, which showed that working memory was related to both recognition and source-monitoring accuracy. Ruffman et al. (2001) proposed that working memory plays a general role in memory ability that applies to recognition as well as source monitoring, rather than a differentiated effect on source monitoring alone.

It was hypothesized that children with better inhibitory control would be more accurate at source monitoring because they would be able to inhibit information from competing sources, and there was moderate support for this hypothesis. Although we did find that inhibitory control was positively correlated with source accuracy, interestingly, inhibitory control was not a significant predictor of source-monitoring accuracy in the regression analysis. The lack of variability in inhibitory control scores may have contributed to the non-significant findings in analyses with these variables. This issue is discussed further in the limitation section.

Similar to our results, Ruffman et al. (2001) and Melinder et al. (2006) found relationships between inhibitory control and some types of source-monitoring tasks, but not others. Ruffman et al. (2001) exposed children to audio and video stories, and showed a significant correlation between a Stroop task and source questions about details that happened in the video or in neither source, but no relationship to questions about details that happened in the audio or both sources. Melinder et al. (2006) found that while inhibitory control was a significant predictor of suggestibility, it was not predictive of source monitoring. Thus, while the inhibitory control-source monitoring relationship is theoretically plausible and evidence for the relationship is present in the literature, it is neither clear nor overwhelming.

### The contributions of executive function in the serial and parallel conditions

One possible explanation for differences in results between various studies is variations in the way the source tasks are presented. We expected that there would be differences in the relationship between executive function and source monitoring as a function of retrieval strategy, and indeed there were differentiated relationships between components of executive function and source monitoring. Specifically, working memory was important for both tasks, but inhibitory control was only related to source monitoring with a parallel approach.

In the serial interview condition, children were asked to think about only one source at a time. Clearly working memory would be involved in remembering this rule, but we also predicted that inhibitory control would be important as children were required to inhibit competing information from the other source. As well, they would have to inhibit simple familiarity-based processes as they had to “filter” their memories, including details in their report only if a remembered detail was *also* accompanied by a determination of the target source, rather than anything that was at the activities.

Of relevance to this null result is the fact that source accuracy was lower in the serial condition compared to the parallel condition. Thus, it is possible that children in the serial condition were simply remembering information with less regard to source than their counterparts (i.e., failing to “filter” through source). While all age groups scored above chance in source monitoring, it is possible that children in the serial condition were simply engaging in less source reasoning than those in the parallel condition. Therefore, an inhibitory control-source monitoring relationship would be less apparent in the serial condition if children were not engaging as extensively in source monitoring processes.

Scores on the Stroop task were related to source monitoring accuracy in the parallel condition. This is consistent with a previous study showing that inhibitory control was related to resistance to suggestions about a series of repeated events (Roberts and Powell, 2005). Although we had originally anticipated that inhibitory control would play a stronger role in the serial condition, where children were required to inhibit details from other sources, it is clear that inhibition serves a useful function when children are making decisions about several competing sources as well. In our parallel processing task, children sorted cards between several different boxes, and the presentation of competing source options may have required inhibitory control as well as working memory.

Perhaps the fact that young children are not proficient in inhibitory control may underlie their lack of spontaneously recalling sources in parallel. This is supported by the finding that the younger age groups (4- to 5 and 6-year-olds) who were provided with a “compare and contrast” strategy improved their source monitoring relative to those practicing a serial retrieval strategy. The relationship might also be bidirectional so that engaging in a parallel retrieval strategy necessitates an improvement in inhibitory control.

Young children can monitor self-other sources before they can monitor two internally generated events (e.g., imagined and dreamt events; Foley et al., 1983; Foley and Johnson, 1985). Similarly, young children are disproportionately less able to monitor sources that are similar compared to older children and adults (Lindsay et al., 1991; Roberts and Blades, 1999). These findings demonstrate clearly that the demands of source-monitoring tasks have diverse influences on accuracy resulting in different developmental patterns. Thus, it may be fruitful to consider what factors contribute to task difficulty and interweave this with investigations of inhibitory control-source monitoring relationships. Careful study of the characteristics of source tasks and how they influence the role of the cognitive factors involved in source monitoring is a necessary step to better understanding the executive underpinnings of source-monitoring development.

### Serial vs. parallel source accuracy

We hypothesized that there would be differences in source accuracy when children recalled sources serially vs. in parallel, with the parallel condition showing an advantage over the serial condition. This was true for the two younger age groups but not for the older children. Accuracy scores were very similar across conditions for the 7- to 8-year-olds, and there was a large difference between the scores for 4- and 5-year-olds in the two conditions; the children in the serial condition demonstrated poor source-monitoring abilities, and the children in the parallel condition improved by 15%, bringing their performance close to that of the 7- to 8-year-olds.

When young children considered both sources at the same time during the decision-making process, they monitored source more carefully and benefitted from the facilitation of a comparison strategy. In the serial condition children were provided with the opportunity to spontaneously use a strategy, but were not assisted with comparing sources. We believe that differences between serial and parallel retrieval strategies were not evident for the 7- to 8-year-old group because these children were able to spontaneously engage in parallel retrieval of sources without the interviewer facilitating such a strategy. Developmentally, it is around this time that children are close to adult proficiency in some types of source recall (Roberts, 2002).

### Practical implications

The results of this study have implications for educational and forensic contexts. Younger children may be preoccupied with absorbing content rather than source information because it is more important for young children to build up a knowledge base, and only in later years concern themselves with recalling where information came from (Roberts and Powell, 2005). This lack of attention to source has been well documented in several different areas of cognitive development (e.g., Gopnik and Graf, 1988). In contrast, older children who have built up a significant (though by no means complete) knowledge of the world have more cognitive resources available for attending to the sources of information. Indeed, as children become habitual internet-users and the availability of information grows, making judgments about the credibility of information from different sources will serve children well.

These findings are also relevant to forensic investigations involving children. For example, many children in abuse investigations are asked to provide specific information about an alleged incident, which requires them to distinguish between instances because child abuse often occurs more than once (Ceci and Bruck, 1993). Children might confuse details from similar events that happened a long time ago because they confuse the origins of events (Roberts and Blades, 1999). Developing techniques that compensate for young children's still-developing proficiency in executive function and source monitoring is a difficult but especially important challenge.

Investigators may be able to encourage children to directly compare sources and think carefully about multiple instances before deciding in which event a detail occurred in order to increase source accuracy. Most children with experiences of repeated abuse will have built up a script and may not realize the importance of reporting details specific to just one instance, so drawing children's attention to sources in this way may facilitate source monitoring performance. For example, Brubacher et al. (2011) have found that giving children practice in talking about occurrences of a repeated event (e.g., swimming lessons) improved their reports when asked to discuss target instances of another repeated event. The fact that the parallel retrieval strategy in this study improved source monitoring through the task procedure alone without a separate training procedure makes this technique ideal for investigators, as it requires few resources to employ. However, more research on the effectiveness of this technique is needed before generalizations are made.

### Limitations

A limitation of the current study was that the inhibitory control scores showed evidence of ceiling effects. Inhibitory control shows rapid improvements in early childhood, with the largest improvements in tasks like Luria's Hand Game around age 4 (Best and Miller, 2010). Therefore, it would be expected that 6- to 8-year-olds would have similarly high scores on these tasks, whereas the 4- to 5-year-olds would not have scores as high as the older children. Although the scores in inhibitory control tasks were quite high in this study, the relationships with age and working memory were significant, so it was not the case that variability was so restricted that it was not possible to find significant relationships with other variables. Reaction time data were not available in our study, but this type of data might be considered more useful for future research as it may show more variability and be less susceptible to ceiling effects.

Another limitation of this study is that several aspects of the methodology may have reduced the demands of executive function in the current source-monitoring tasks. The boxes labeled with the source names may have reduced the need for working memory because children were not required to hold the possible source options in mind as they thought about details in the way they would have been if the questions were asked verbally. As well, our two-step memory task may have reduced demands on executive function compared to a task where recognition and source were combined using “Story,” “Real-Life,” and “Neither” options, because in these tasks children are required to think about whether they saw a detail and what source it was in at the same time. However, although this two-step procedure may be less cognitively demanding overall, it allowed for an investigation of the relationships of executive function to recognition and source-monitoring accuracy separately.

## Conclusion

This study adds evidence to the growing body of literature on the underlying mechanisms of source-monitoring development and, overall, these findings have illustrated the relations between executive function and source-monitoring accuracy. Working memory seems to be necessary for source monitoring in general, even when the exact nature of the task varies. The role of inhibitory control in source monitoring is less clear, although inhibitory control was positively correlated with memory and source accuracy. Further research is necessary to clarify mixed results about the contributions of working memory and inhibitory control to source-monitoring performance in previous research. Although this study contributes to the body of literature on this topic, it does not ultimately provide a definitive answer to that question.

The results of this research address both practical and theoretical questions about what interview strategies are most helpful for children when they are making source-monitoring decisions. Knowing more about the cognitive prerequisites for source monitoring helps determine what to expect from children of different ages and cognitive abilities. An important area for future research is the investigation of how task difficulty affects the relationship between executive function and source monitoring.

## Author contributions

Both authors contributed ideas to the development of the project. Data was collected, coded and analyzed by Becky Earhart in collaboration with a team of research assistants. Both authors made significant contributions to the writing of this manuscript.

### Conflict of interest statement

The authors declare that the research was conducted in the absence of any commercial or financial relationships that could be construed as a potential conflict of interest.
